# The randomness that shapes our DNA

**DOI:** 10.7554/eLife.41491

**Published:** 2018-10-09

**Authors:** Kelley Harris

**Affiliations:** Department of Genome SciencesUniversity of WashingtonSeattleUnited States

**Keywords:** genomic diversity, natural selection, GC-biased gene conversion, recombination, demography, evolution, Human

## Abstract

Just 5% of the human genome is subject to neutral evolution, but this process remains central to understanding the history of human migration across the Earth.

**Related research article** Pouyet F, Aeschbacher S, Thiéry A, Excoffier L. 2018. Background selection and biased gene conversion affect more than 95% of the human genome and bias demographic inferences. *eLife* **7**:e36317. doi: 10.7554/eLife.36317

Darwin famously thought of evolution as a force for creation and improvement. Evolution, in his eyes, was synonymous with natural selection and survival of the fittest – a process that optimizes life to thrive in its environment and produce greater numbers of offspring. A century later, however, the great population geneticist Motoo Kimura challenged this view with his 'neutral theory' of evolution (Kimura, 1968). While the neutral theory was more difficult to understand than Darwin's approach, and hence did not capture the public imagination in the same way, it provided the key insight that helped evolution to make sense in the light of molecular biology.

Unlike Darwin, Kimura knew that 'descent with modification' is caused by the transmission of DNA from parents to offspring. When DNA is copied faithfully, children tend to resemble their parents, but copying mistakes and mutagens occasionally create small changes in the DNA sequence, known as genetic variants. Over millions of years, these mutations provided the raw material for natural selection to make a human out of an ape.

It may be tempting to analyze each genetic difference between humans and chimps and wonder why natural selection might have chosen exactly these mutations to build the human phenotype. But Kimura’s theory suggests that this would be a fool’s errand because the vast majority of mutations have no effect on fitness whatsoever. If most mutations fail to alter the fitness of an offspring who carries them, random chance will dictate whether they survive or die. That is, say we compared a human genome and a chimp genome and discovered millions of differences between them: the neutral theory would predict that most of these differences accumulated by chance and that only a tiny fraction gave some kind of fitness advantage to the ancestors of modern humans.

But how much of the genome does indeed evolve neutrally? Although the neutral theory turned 50 this year, this seemingly basic question is still a topic of hot debate (Rands et al., 2014; Graur, 2017). Even the question of what it means for a mutation to evolve neutrally is more complicated than Kimura could have imagined in 1968. Now, in eLife, Fanny Pouyet, Simon Aeschbacher, Alexandre Thiéry and Laurent Excoffier revisit these questions (Pouyet et al., 2018).

Unlike adaptive mutations, which spread through populations as fast as their fitness advantages can carry them, neutral mutations diffuse at a slow, steady rate that is easy to model mathematically (Fisher, 1930). In theory, it is possible to: i) sample DNA sequences from humans worldwide; ii) count and compare the neutral mutations that can be found on multiple continents, one continent, and one individual; iii) use this information to reconstruct details about human migration across the globe (Gutenkunst et al., 2009). In practice, however, it can be hard to deduce whether a given mutation is evolving neutrally or not. When variation under selection is misclassified as neutral and used to study past migrations and changes in population size, the results can be misleading (Ewing and Jensen, 2016; Schrider et al., 2016).

In an ambitious undertaking, Pouyet et al. – who are based at the University of Bern, the Swiss Institute of Bioinformatics and the University of Zurich – discovered how much of the human genome can really be used for this style of demographic analysis. Their results showed that only 5% of the genome is truly evolving neutrally, with the remaining 95% being affected by some kind of natural selection. Superficially, this might seem like a death knell for the neutral theory, but it is nothing of the kind. To understand why, we have to revisit the question of what it means to evolve neutrally.

A mutation will not evolve neutrally if it provides a direct fitness advantage, but the converse does not apply. A mutation can appear to evolve non-neutrally if it is merely located close to a mutation that affects fitness. In sexual organisms like humans, each child inherits DNA from its parents in big, continuous chunks. Even distant cousins will share large chunks of DNA that were inherited from recent common ancestors. If that part of DNA happens to contain a beneficial mutation, hundreds of nearby mutations might hitchhike along for the ride as the beneficial DNA spreads quickly through a population (Smith and Haigh, 1974; Charlesworth et al., 1993). These hitchhikers confer no fitness advantage but, equally, they do not behave the way we expect neutral mutations to do.

Because of this hitchhiking effect, Pouyet et al. advise that genetic variation does not behave neutrally in those regions of the genome that have the lowest recombination rates. (Recombination is the process that stitches together chromosomes from a father and a mother to create a child’s genome.) In regions with high recombination rates, on the other hand, neutral mutations are quickly separated from nearby mutations that are under selection. This makes hitchhiking less of a concern and neutrality a better model.

However, there is one other, somewhat strange, process that we must take into account: biased gene conversion is a process that causes a large fraction of genetic variation to behave non-neutrally, even in regions with high recombination rates (Galtier et al., 2001). It is a quirk of mammalian biochemistry that biased gene conversion makes it slightly more likely for G–C base pairs to be passed down to offspring than A–T base pairs, even when the latter might enhance fitness. As a result, a mutation from A to C may behave like it is beneficial when it is in fact neutral or slightly deleterious. Pouyet et al. suggest that only mutations from G to C, or from A to T, can be truly considered to be neutrally evolving. Together with the restriction to regions with high recombination rates, this rule narrows down the fraction of the genome that is neutrally evolving to 5%.

This amount may seem paltry compared to Kimura’s assertion that neutrality dominates the genome, but it nevertheless contains hundreds (possibly thousands) of mutations, depending on how many human genomes one is looking at simultaneously. These certifiably neutral mutations should prove to be a great resource for future analyses of human demographic history, as it may no longer be defensible to use the whole human genome to make inferences about human migration. It remains to be seen how much of what we think we know about human history has been thrown off by non-neutral evolutionary forces.
